# Supplementary material for: Low-Carbohydrate and Low-Fat Diet with Metabolic-Dysfunction-Associated Fatty Liver Disease
Source: Nutrients. 2023 Nov 13;15(22):4763. doi: 10.3390/nu15224763 (PMC10674227; doi:10.3390/nu15224763)
Supplement: Supplementary file 1 [file nutrients-15-04763-s001.zip › nutrients-2687959-supplementary.pdf]

Supplementary Online Content

Low-carbohydrate and Low-fat Diet with metabolic-dysfunction-associated fatty liver disease

Chengxiang Hu, Rong Huang

Supplemental Methods

Supplementary Figure S1. Flowchart of selecting participants in the analysis.

Supplementary Table S1. Criteria for Determining the Low-Carbohydrate-Diet Scores

Supplementary Table S2. Criteria for Determining the Low-Fat-Diet Scores

Supplementary Table S3. Correlation matrix between diet scores

Supplementary Figure S2. Odds ratio (ORs) of MAFLD per 5 points increases in healthy LCD score and healthy LFD score by Subgroups in NHANES (2017-2018).

Supplementary Figure S3. Odds ratio (ORs) of MAFLD per 5-points increase in unhealthy LCD score and unhealthy LFD score by Subgroups in NHANES (2017-2018). LCD, low-carbohydrate diet; LFD, low-fat diet; MAFLD, metabolic dysfunction associated fatty liver disease.

Supplementary Table S4. Association between LCD scores, LFD scores and MAFLD without hepatitis B or C virus.

Supplementary Table S5. Association between LCD scores, LFD scores and MAFLD without history of heart disease.

Supplementary Table S6. Association between specific macronutrients and MAFLD.

Reference

Supplemental Methods

Assessment of Dietary Intake

NHANES has been implemented since 1999 using a complex, stratified, clustered multistage probability sampling design, which dietary data was evaluated applying 24-h recall, and twice collections would be carried out about 3-10 days. First 24-h recall was conducted in the Mobile Examination Center and the second by a telephone interview, even the new 5-step Automated Multiple-Pass Method was introduced to enhance the accuracy of dietary recall. In this study, we harmonized the US Department of Agriculture (USDA) Food Patterns Equivalents Database and the USDA Food and Nutrition Database for Dietary Studies (FNDDS) to assess macronutrient in detail.(1) Briefly, the major food groups offering high-quality carbohydrate were whole grains, whole fruit, legumes, and non-starchy vegetables, and offering low-quality carbohydrate were refined grains, added sugar, fruit juice, potato, other starchy vegetables and residual carbohydrates.(2) We defined animal protein as protein from fish/seafood, meat, cured meat, organ, poultry, egg, dairy products, and other animal sources and plant protein as protein from whole grains, refined grains, nuts, legumes, soy, and other plant sources. (3) To measure diet exposure perfectly, average consumption was utilized for assessing dietary intake.

Assessment of MAFLD

MAFLD is a multisystem disease. Besides hepatic steatosis, individuals with MAFLD should suffer from at least one of the following three conditions: i) overweight or obesity(Body Mass Index (BMI) ≥25 kg/m^2); ii) presence of diabetes mellitus (fasting glucose ≥7.0 mmol/L or hemoglobin A1c ≥6.5% or antidiabetic medications; iii) at least 2 metabolic risk abnormalities, including (a) Waist circumference(WC) ≥ 102 in men and 88 cm in women;(b) blood pressure ≥ 130/85 mmHg or specific drug treatment; (c) Plasma triglycerides ≥ 150 mg/dL (≥1.70 mmol/L) or specific drug treatment; (d) Plasma HDL-cholesterol < 1.0 mmol/L for male and < 1.3mmol/L for female or specific drug treatment; (e) prediabetes (fasting glucose 5.6-6.9 mmol/L or hemoglobin A1c 5.7%-6.4%; (f) homeostasis model assessment of insulin resistance score ≥2.5; (g) Plasma high-sensitivity C-reactive protein level > 2 mg/L.

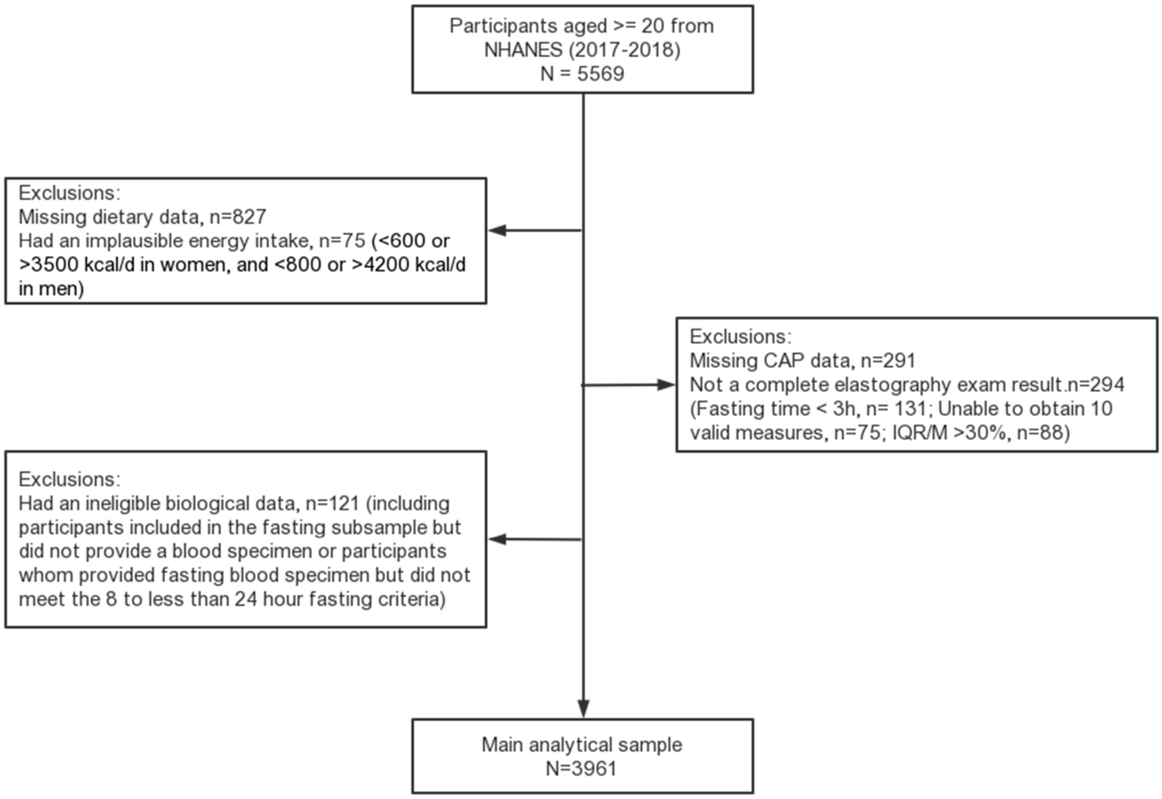

Supplementary Figure S1. Flowchart of selecting participants in the analysis. CAP, Controlled Attenuation Parameter; NHANES, National Health and Nutrition Examination Survey.

Supplementary Table S1. Criteria for Determining the Low-Carbohydrate-Diet Scores

Points	Overall LCD Score			Unhealthy LCD Score			Healthy LCD Scores		
	Total carbohydrate	Total fat	Total protein	High quality carbohydrate	Saturated fat	Animal protein	Low quality carbohydrate	Unsaturated fat	Plant protein
	Dietary intake, % of total energy intake								
0	< 56.48	< 28.19	< 13.58	> 16.82	< 9.34	< 8.33	> 48.56	< 18.09	< 4.20
1	54.35 - 56.48	28.19 - 29.93	13.58 - 14.37	13.85 - 16.82	9.34 - 10.16	8.33 - 9.13	46.16 - 48.56	18.09 - 19.17	4.20 - 4.51
2	52.99 - 54.34	29.94 - 31.13	14.38 - 14.97	12.04 - 13.84	10.17 - 10.69	9.14 - 9.70	44.43 - 46.15	19.18 - 19.96	4.52 - 4.75
3	51.82 - 52.98	31.14 - 32.11	14.98 - 15.50	10.62 - 12.03	10.70 - 11.16	9.71 - 10.19	42.94 - 44.42	19.97 - 20.64	4.76 - 4.95
4	50.79 - 51.81	32.12 - 33.05	15.51 - 16.01	9.20 - 10.61	11.17 - 11.60	10.20 - 10.67	41.50 - 42.93	20.65 - 21.25	4.96 - 5.14
5	49.62 - 50.78	33.06 - 33.99	16.02 - 16.47	8.03 - 9.19	11.61 - 12.02	10.68 - 11.13	40.29 - 41.49	21.26 - 21.86	5.15 - 5.34
6	48.49 - 49.61	34.00 - 34.92	16.48 - 17.03	6.99 - 8.02	12.03 - 12.45	11.14 - 11.63	38.64 - 40.28	21.87 - 22.52	5.35 - 5.56
7	47.28 - 48.48	34.93 - 36.00	17.04 - 17.58	5.78 - 6.98	12.46 - 12.93	11.64 - 12.22	36.97 - 38.63	22.53 - 23.23	5.57 - 5.81
8	45.73 - 47.27	36.01 - 37.14	17.59 - 18.33	4.42 - 5.77	12.94 - 13.51	12.23 - 12.92	35.15 - 36.96	23.24 - 24.25	5.82 - 6.12
9	43.36 - 45.72	37.15 - 38.90	18.34 - 19.42	3.08 - 4.41	13.52 - 14.35	12.93 - 14.08	32.33 - 35.14	24.26 - 25.58	6.13 - 6.64
10	< 43.36	> 38.90	> 19.42	< 3.08	> 14.35	> 14.08	< 32.33	> 25.58	> 6.64

Abbreviations: LCD, Low-carbohydrate diet.

High-quality carbohydrate was defined as carbohydrate from whole grains, whole fruit, legumes, and non-starchy vegetables. Low-quality carbohydrate was defined as carbohydrate from refined grains, added sugar, fruit juice, potato, other starchy vegetables, and other sources.

Supplementary Table S2. Criteria for Determining the Low-Fat-Diet Scores

Points	Overall LFD Score			Unhealthy LFD Score			Healthy LFD Scores		
	Total fat	Total carbohydrate	Total protein	Unsaturated fat	Low quality carbohydrate	Animal protein	Saturated fat	High quality carbohydrate	Plant protein
	Dietary intake, % of total energy intake								
0	> 38.90	< 43.36	< 13.58	> 25.58	< 32.33	< 8.33	> 14.35	< 3.08	< 4.20
1	37.15 - 38.90	43.36 - 45.72	13.58 - 14.37	24.26 - 25.58	32.33 - 35.14	8.33 - 9.13	13.52 - 14.35	3.08 - 4.41	4.20 - 4.51
2	36.01 - 37.14	45.73 - 47.27	14.38 - 14.97	23.24 - 24.25	35.15 - 36.96	9.14 - 9.70	12.94 - 13.51	4.42 - 5.77	4.52 - 4.75
3	34.93 - 36.00	47.28 - 48.48	14.98 - 15.50	22.53 - 23.23	36.97 - 38.63	9.71 - 10.19	12.46 - 12.93	5.78 - 6.98	4.76 - 4.95
4	34.00 - 34.92	48.49 - 49.61	15.51 - 16.01	21.87 - 22.52	38.64 - 40.28	10.20 - 10.67	12.03 - 12.45	6.99 - 8.02	4.96 - 5.14
5	33.06 - 33.99	49.62 - 50.78	16.02 - 16.47	21.26 - 21.86	40.29 - 41.49	10.68 - 11.13	11.61 - 12.02	8.03 - 9.19	5.15 - 5.34
6	32.12 - 33.05	50.79 - 51.81	16.48 - 17.03	20.65 - 21.25	41.50 - 42.93	11.14 - 11.63	11.17 - 11.60	9.20 - 10.61	5.35 - 5.56
7	31.14 - 32.11	51.82 - 52.98	17.04 - 17.58	19.97 - 20.64	42.94 - 44.42	11.64 - 12.22	10.70 - 11.16	10.62 - 12.03	5.57 - 5.81
8	29.94 - 31.13	52.99 - 54.34	17.59 - 18.33	19.18 - 19.96	44.43 - 46.15	12.23 - 12.92	10.17 - 10.69	12.04 - 13.84	5.82 - 6.12
9	28.19 - 29.93	54.35 - 56.48	18.34 - 19.42	18.09 - 19.17	46.16 - 48.56	12.93 - 14.08	9.34 - 10.16	13.85 - 16.82	6.13 - 6.64
10	< 28.19	> 56.48	> 19.42	< 18.09	> 48.56	> 14.08	< 9.34	> 16.82	> 6.64

Abbreviations: LFD, low-fat diet.

High-quality carbohydrate was defined as carbohydrate from whole grains, whole fruit, legumes, and non-starchy vegetables. Low-quality carbohydrate was defined as carbohydrate from refined grains, added sugar, fruit juice, potato, other starchy vegetables, and other sources.

Supplementary Table S3. Correlation matrix between diet scores.

	Overall LCD Score	Healthy LCD Scores	Unhealthy LCD Score	Overall LFD Score	Healthy LFD Scores	Unhealthy LFD Score
Overall LCD Score	1	-	-	-	-	-
Healthy LCD Scores	0.646	1	-	-	-	-
Unhealthy LCD Score	0.724	0.014	1	-	-	-
Overall LFD Score	-0.609	-0.397	-0.508	1	-	-
Healthy LFD Scores	-0.356	0.348	-0.823	0.594	1	-
Unhealthy LFD Score	-0.388	-0.777	0.127	0.678	-0.113	1

Abbreviations: LCD, low-carbohydrate diet; LFD, low-fat diet.

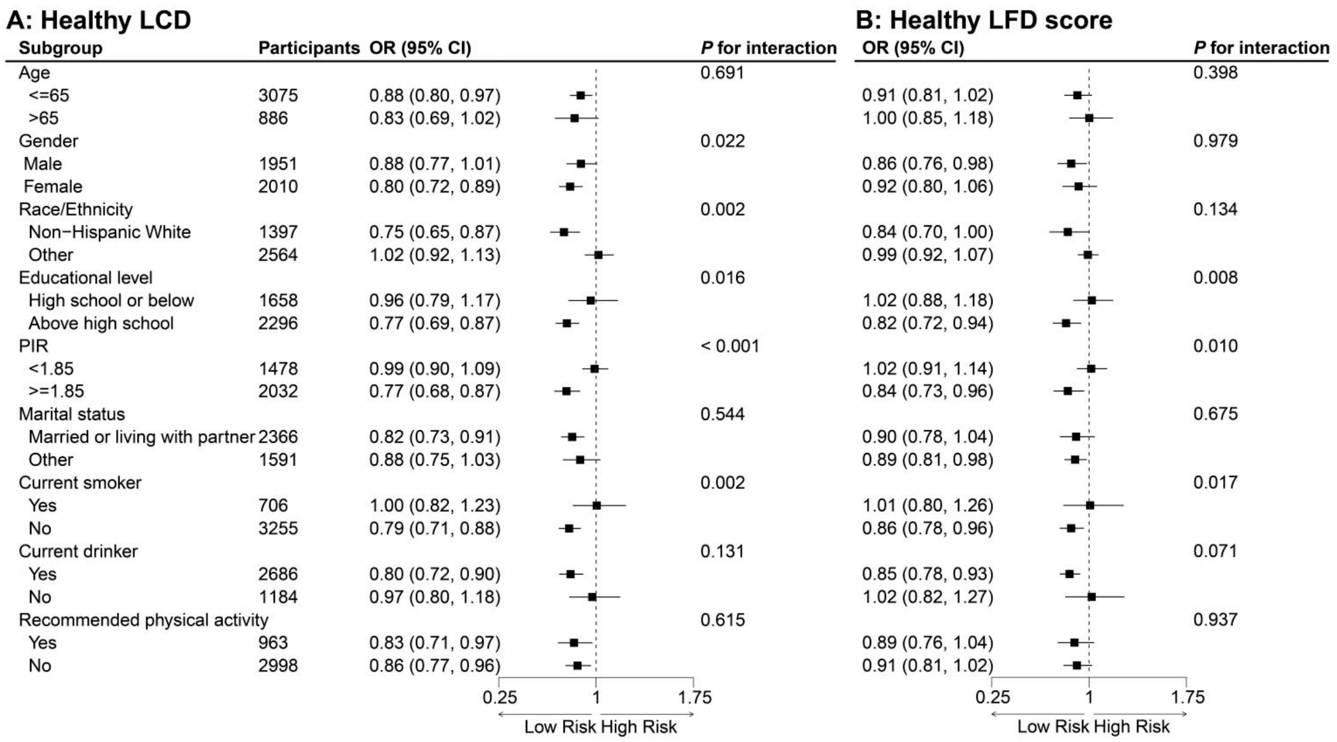

Supplementary Figure S2. Odds ratio (ORs) of MAFLD per 5 points increases in healthy LCD score and healthy LFD score by Subgroups in NHANES (2017-2018).

LCD, low-carbohydrate diet; LFD, low-fat diet; MAFLD, metabolic dysfunction associated fatty liver disease.

Adjusted for age (continuous), gender (male/female), race/ethnicity (non-Hispanic White/other), educational level (High school or below/College or above), marital status (Married or living with partner/other), PIR (<1.85/>=1.85), current smoker (yes/no), current drinker (yes/no), Recommended physical activity (yes/no), total energy intake (continuous).

Missing data in subgroup: educational level (n=7); marital status (n=4); PIR (n=451); current drinker (n=91).

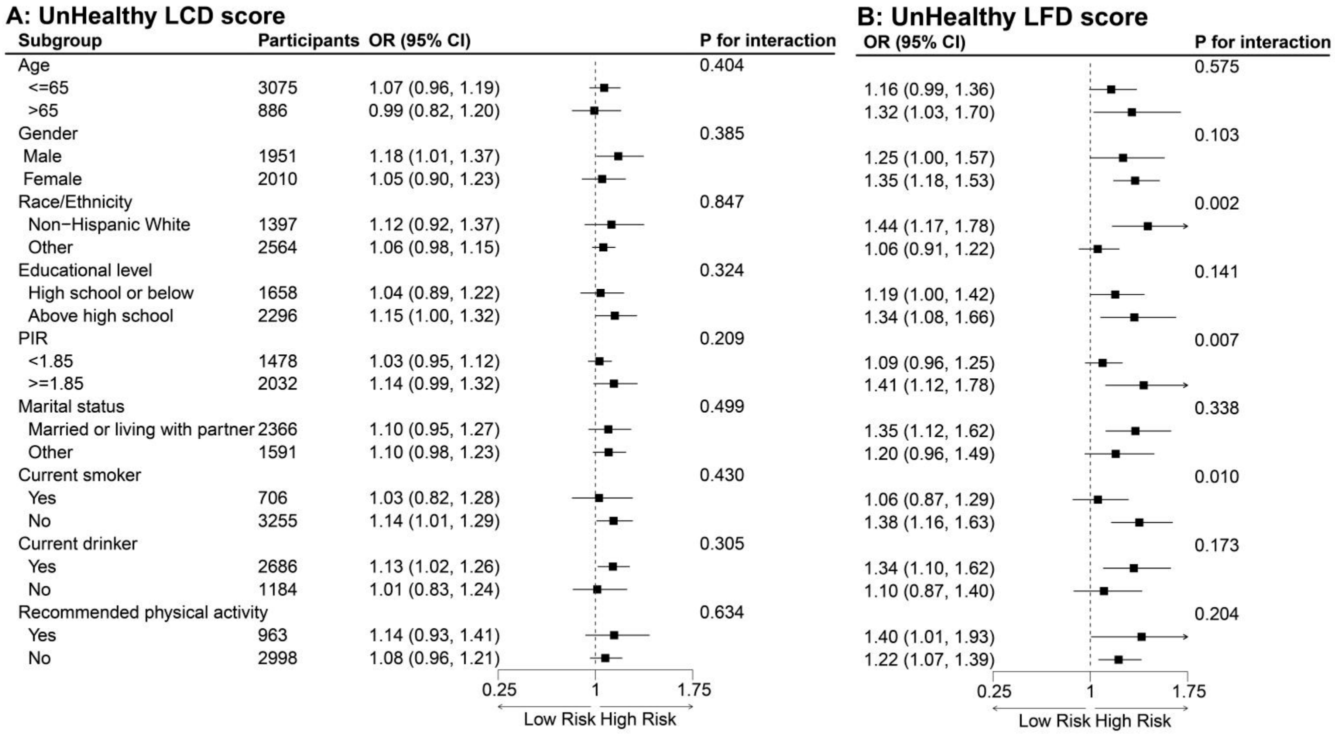

Subgroup	Participants	A: UnHealthy LCD score OR (95% CI)	P for interaction	B: UnHealthy LFD score OR (95% CI)	P for interaction
Age			0.404		0.575
<=65	3075	1.07 (0.96, 1.19)		1.16 (0.99, 1.36)	
>65	886	0.99 (0.82, 1.20)		1.32 (1.03, 1.70)	
Gender			0.385		0.103
Male	1951	1.18 (1.01, 1.37)		1.25 (1.00, 1.57)	
Female	2010	1.05 (0.90, 1.23)		1.35 (1.18, 1.53)	
Race/Ethnicity			0.847		0.002
Non−Hispanic White	1397	1.12 (0.92, 1.37)		1.44 (1.17, 1.78)	
Other	2564	1.06 (0.98, 1.15)		1.06 (0.91, 1.22)	
Educational level			0.324		0.141
High school or below	1658	1.04 (0.89, 1.22)		1.19 (1.00, 1.42)	
Above high school	2296	1.15 (1.00, 1.32)		1.34 (1.08, 1.66)	
PIR			0.209		0.007
<1.85	1478	1.03 (0.95, 1.12)		1.09 (0.96, 1.25)	
>=1.85	2032	1.14 (0.99, 1.32)		1.41 (1.12, 1.78)	
Marital status			0.499		0.338
Married or living with partner	2366	1.10 (0.95, 1.27)		1.35 (1.12, 1.62)	
Other	1591	1.10 (0.98, 1.23)		1.20 (0.96, 1.49)	
Current smoker			0.430		0.010
Yes	706	1.03 (0.82, 1.28)		1.06 (0.87, 1.29)	
No	3255	1.14 (1.01, 1.29)		1.38 (1.16, 1.63)	
Current drinker			0.305		0.173
Yes	2686	1.13 (1.02, 1.26)		1.34 (1.10, 1.62)	
No	1184	1.01 (0.83, 1.24)		1.10 (0.87, 1.40)	
Recommended physical activity			0.634		0.204
Yes	963	1.14 (0.93, 1.41)		1.40 (1.01, 1.93)	
No	2998	1.08 (0.96, 1.21)		1.22 (1.07, 1.39)	

Supplementary Figure S3. Odds ratio (ORs) of MAFLD per 5-points increase in unhealthy LCD score and unhealthy LFD score by Subgroups in NHANES (2017-2018). LCD, low-carbohydrate diet; LFD, low-fat diet; MAFLD, metabolic dysfunction associated fatty liver disease.

Adjusted for age (continuous), gender (male/female), race/ethnicity (non-Hispanic White/other), educational level (High school or below/College or above), marital status (Married or living with partner/other), PIR (<1.85/≥1.85), current smoker (yes/no), current drinker (yes/no), Recommended physical activity (yes/no), total energy intake (continuous).

Missing data in subgroup: educational level (n=7); marital status (n=4); PIR (n=451); current drinker (n=91).

Supplementary Table S4. Association between LCD scores, LFD scores and MAFLD without hepatitis B or C virus.

Association between LCD scores, LFD scores and MAFLD

	Tertiles of diet scores			*P* for trend	Per 5-points increase
	Tertile 1	Tertile 2	Tertile 3		
Overall LCD score					
Median score (IQR)	7 (4-9)	16 (13-17)	24 (22-27)		
Cases/participants, n/n	768/1396	712/1242	709/1224		
Model 1	Reference	0.96 (0.70, 1.31)	0.84(0.63, 1.31)	0.237	0.94 (0.87, 1.01)
Model 2	Reference	0.95 (0.70, 1.29)	0.88 (0.65, 1.22)	0.439	0.95 (0.87, 1.04)
Unhealthy LCD score					
Median score (IQR)	8 (5-11)	16 (14-17)	22 (20-24)		
Cases/participants, n/n	766/1388	702/1230	721/1244		
Model 1	Reference	**1.37 (1.12, 1.66)**	**1.39(1.05, 1.83)**	**0.020**	**1.11 (1.01, 1.20)**
Model 2	Reference	1.32 (1.00, 1.73)	**1.43 (1.01, 2.02)**	**0.044**	**1.12 (1.00, 1.25)**
Healthy LCD score					
Median score (IQR)	9 (6-11)	16 (14-17)	22 (20-24)		
Cases/participants, n/n	799/1437	692/1177	698/1248		
Model 1	Reference	**0.85 (0.68, 1.06)**	**0.59 (0.44, 0.80)**	**0.002**	**0.84 (0.78, 0.91)**
Model 2	Reference	0.82 (0.63, 1.07)	**0.60 (0.42, 0.87)**	**0.008**	**0.85 (0.77, 0.93)**
Overall LFD score					
Median score (IQR)	9 (7-11)	15 (14-17)	21 (20-23)		
Cases/participants, n/n	801/1414	710/1271	678/1177		
Model 1	Reference	1.16 (0.92, 1.47)	**1.23 (0.92, 1.67)**	**0.131**	1.07 (0.97, 1.19)
Model 2	Reference	1.21 (0.92, 1.58)	1.28 (0.86, 1.91)	0.179	1.10 (0.96, 1.26)
Unhealthy LFD score					
Median score (IQR)	10 (8-12)	16 (15-17)	21 (20-23)		
Cases/participants, n/n	871/1533	729/1287	589/1042		
Model 1	Reference	**1.53 (1.26, 1.86)**	**1.72 (1.26, 2.34)**	**0.001**	**1.26 (1.11, 1.44)**
Model 2	Reference	**1.59(1.23, 2.05)**	**1.82 (1.21, 2.72)**	**0.004**	**1.29 (1.09, 1.52)**
Healthy LFD score					
Median score (IQR)	8 (5-10)	15 (13-17)	23 (21-26)		
Cases/participants, n/n	773/1349	715/1233	701/1280		
Model 1	Reference	0.87 (0.72, 1.06)	**0.62 (0.49, 0.78)**	**0.001**	**0.87(0.81, 0.94)**
Model 2	Reference	0.90 (0.70, 1.15)	**0.63 (0.47, 0.85)**	**0.006**	**0.88 (0.80, 0.98)**

Abbreviations: LCD, low-carbohydrate diet; LFD, low-fat diet; MAFLD, metabolic dysfunction associated fatty liver disease.

Model 1: Adjusted for age (continuous), gender (male/female), race/ethnicity (non-Hispanic White/ other).

Model 2: Adjusted for age (continuous), gender (male/female), race/ethnicity (non-Hispanic White/other), educational level (High school or below/College or above), marital status (Married or living with partner/other), PIR (<1.85/≥1.85), current smoker (yes/no), current drinker (yes/no), Recommended physical activity (yes/no), total energy intake (continuous).

Supplementary Table S5. Association between LCD scores, LFD scores and MAFLD without history of heart disease.

	Tertiles of diet scores			*P* for trend	Per 5-points increase
	Tertile 1	Tertile 2	Tertile 3		
Overall LCD score					
Median score (IQR)	7 (4-9)	16 (13-17)	24 (22-27)		
Cases/participants, n/n	677/1274	639/1152	632/1115		
Model 1	Reference	0.98 (0.72, 1.34)	0.94 (0.71, 1.25)	0.661	0.96 (0.89, 1.04)
Model 2	Reference	0.95 (0.70, 1.29)	0.97 (0.71, 1.33)	0.855	0.96 (0.88, 1.06)
Unhealthy LCD score					
Median score (IQR)	9 (5-11)	16 (14-17)	22 (20-24)		
Cases/participants, n/n	685/1273	633/1135	630/1133		
Model 1	Reference	1.30 (1.01, 1.67)	**1.36 (1.01, 1.84)**	**0.050**	**1.11 (1.01, 1.22)**
Model 2	Reference	1.22 (0.88, 1.68)	1.37(0.94, 2.02)	0.101	1.12 (0.99, 1.27)
Healthy LCD score					
Median score (IQR)	9 (6-11)	16 (14-17)	22 (20-24)		
Cases/participants, n/n	707/1316	623/1102	618/1123		
Model 1	Reference	0.83 (0.60, 1.14)	**0.67 (0.50, 0.89)**	**0.010**	**0.87 (0.80, 0.94)**
Model 2	Reference	0.74 (0.52, 1.04)	**0.65 (0.45, 0.92)**	**0.014**	**0.85 (0.77, 0.94)**
Overall LFD score					
Median score (IQR)	9 (7-11)	15.5 (14-17)	21 (20-22)		
Cases/participants, n/n	709/1298	630/1172	609/1071		
Model 1	Reference	1.10 (0.81, 1.52)	1.17 (0.89, 1.55)	0.267	1.05 (0.94, 1.18)
Model 2	Reference	1.14(0.79, 1.63)	1.23 (0.84, 1.81)	0.282	1.07 (0.93, 1.24)
Unhealthy LFD score					
Median score (IQR)	10 (8-12)	16 (15-17)	21 (20-23)		
Cases/participants, n/n	784/1411	642/1177	522/953		
Model 1	Reference	**1.43 (1.14, 1.81)**	**1.61 (1.16, 2.24)**	**0.004**	**1.22 (1.06, 1.40)**
Model 2	Reference	**1.49 (1.12, 1.97)**	**1.76 (1.15, 2.72)**	**0.009**	**1.25 (1.05, 1.49)**
Healthy LFD score					
Median score (IQR)	8 (5-10)	15 (13-17)	23 (21-26)		
Cases/participants, n/n	675/1232	641/1142	632/1167		
Model 1	Reference	0.87(0.70, 1.08)	**0.64 (0.49, 0.84)**	**0.005**	**0.88 (0.81, 0.96)**
Model 2	Reference	0.87 (0.66, 1.14)	**0.64 (0.44, 0.92)**	**0.023**	**0.88 (0.79, 0.99)**

Abbreviations: LCD, low-carbohydrate diet; LFD, low-fat diet; MAFLD, metabolic dysfunction associated fatty liver disease.

Model 1: Adjusted for age (continuous), gender (male/female), race/ethnicity (non-Hispanic White/ other).

Model 2: Adjusted for age (continuous), gender (male/female), race/ethnicity (non-Hispanic White/other), educational level (High school or below/College or above), marital status (Married or living with partner/other), PIR (<1.85/≥1.85), current smoker (yes/no), current drinker (yes/no), Recommended physical activity (yes/no), total energy intake (continuous).

Supplementary Table S6. Association between specific macronutrients and MAFLD.

	Tertiles of macronutrients consumption of total energy intake			Per 3% energy increment of total intake increase
	Tertile 1	Tertile 2	Tertile 3	
Total carbohydrate	Reference	1.02 (0.75, 1.40)	1.16 (0.84, 1.61)	1.04 (0.96, 1.12)
High carbohydrate	Reference	0.84 (0.62, 1.13)	**0.67 (0.48, 0.95)**	**0.90 (0.82, 0.98)**
Low carbohydrate	Reference	1.32 (0.91, 1.90)	**1.58 (1.20, 2.08)**	**1.10 (1.03, 1.17)**
Total fat	Reference	0.88 (0.69, 1.13)	0.79 (0.58, 1.07)	0.96 (0.87, 1.06)
Unsaturated fat	Reference	0.82 (0.59, 1.14)	0.75 (0.55, 1.03)	0.89 (0.78, 1.01)
Saturated fat	Reference	1.02 (0.84, 1.25)	1.15 (0.9, 1.46)	1.08 (0.88, 1.33)
Total protein	Reference	0.81 (0.63, 1.06)	0.96 (0.66, 1.4)	0.96 (0.77, 1.20)
Animal protein	Reference	0.97 (0.73, 1.29)	1.11 (0.74, 1.67)	1.04 (0.84, 1.28)
Plant protein	Reference	1.16 (0.76, 1.76)	0.85 (0.67, 1.08)	**0.67 (0.47, 0.94)**

Adjusted for age (continuous), gender (male/female), race/ethnicity (non-Hispanic White/other), educational level (High school or below/College or above), marital status (Married or living with partner/other), PIR (<1.85/≥1.85), current smoker (yes/no), current drinker (yes/no), Recommended physical activity (yes/no), total energy intake (continuous).

References

1. Blanton CA, Moshfegh AJ, Baer DJ, Kretsch MJ. The USDA automated multiple-pass method accurately estimates group total energy and nutrient intake. J Nutr 2006;136:2594-2599.

2. Drewnowski A, Maillot M, Vieux F. Multiple metrics of carbohydrate quality place starchy vegetables alongside non-starchy vegetables, legumes, and whole fruit. Front Nutr 2022;9:867378.

3. Pasiakos SM, Agarwal S, Lieberman HR, Fulgoni VL, 3rd. Sources and amounts of animal, dairy, and plant protein intake of US adults in 2007-2010. Nutrients 2015;7:7058-7069.
